# A comprehensive diagnostic scheme of morphological combined molecular methylation under bronchoscopy

**DOI:** 10.3389/fonc.2023.1133675

**Published:** 2023-04-27

**Authors:** Jinze Zhang, Haoran Huang, Fan Yu, Yuanyuan Bian, Rui Wang, Hui Liu, Saisai Kang, Bin She, Zhihua Shi

**Affiliations:** ^1^ Department of Thoracic Surgery, The Fourth Hospital of Hebei Medical University, Shijiazhuang, China; ^2^ Department of Academic Development, Tellgen Corporation, Shanghai, China; ^3^ The Cancer Center, The Fourth Hospital of Hebei Medical University, Shijiazhuang, China

**Keywords:** lung cancer, DNA methylation, SHOX2, RASSF1A, bronchoscopy

## Abstract

Methylated *SHOX2* and *RASSF1A* genes are potential biomarkers for lung cancer diagnosis. Therefore, we explored the role of methylation detection combined with morphological bronchoscopic evaluation for lung cancer diagnosis. Bronchoscopy, methylation outcome, and pathological data were collected from 585 patients with lung cancer and 101 controls. The methylation status of the *SHOX2* and *RASSF1A* genes were detected using real-time polymerase chain reaction quantification. Further, the sensitivity and area under the receiver operating characteristic curve of the three methods were analyzed. Among 686 patients, 57.1% had new lesions detected through bronchoscopy and 93.1% of these patients were diagnosed with malignant tumors. Besides, 42.9% of patients had no visible changes under bronchoscopy but there were still 74.8% of them diagnosed with malignant tumors. Bronchoscopy revealed that lung adenocarcinoma, lung squamous cell carcinoma, and small cell lung cancer mainly occurred in the upper and middle lobes. The sensitivity and specificity of methylation detection were 72.8% and 87.1% (vs. cytology 10.4% & 100%), respectively. Therefore, methylated *SHOX2* and *RASSF1A* genes may be promising tumor markers in lung cancer diagnosis. Methylation detection can be an excellent supplementary tool for cytological diagnosis and, combined with bronchoscopy, could form a more effective diagnostic process.

## 1 Introduction

Lung cancer is responsible for more deaths than any other malignancy and is the most common cancer in men and women, representing approximately 20% of all cancer diagnoses in China (1). Lung cancer is a malignant tumor that originates from the bronchial mucosa and alveolar epithelia. Exfoliated tumor cells and metabolites can enter the alveoli directly. Central bronchogenic carcinomas are lung cancers that occur in the main and segmental bronchia and comprise mostly lung squamous cell carcinoma (LUSC) and small cell lung cancer (SCLC). Bronchoscopy is an effective diagnostic tool. Therefore, bronchial flushing fluid (BFF) and bronchoalveolar lavage fluid (BALF) aid in the early diagnosis of respiratory diseases. Currently, bronchoscopy is usually performed for patients with suspected lung cancer, and BFF/BALF samples are obtained for cytological diagnosis. Therefore, BFF/BALF samples are important for early lung cancer diagnosis. However, human factors significantly affect the cytological diagnosis of BFF/BALF, and its diagnostic sensitivity is low (2). Hence, developing a high-sensitivity BFF/BALF analysis method is important. In recent years, gene detection technology has advanced rapidly. Gene detection of tumor biomarkers has high sensitivity and specificity and is widely used in clinical diagnosis, treatment, and prognosis assessment (3). Detecting genetic biomarkers in BFF/BALF may aid in the early diagnosis of lung cancer.

DNA methylation is critical for regulating gene expression and maintaining cell characteristics. Similarly, epigenetic changes in DNA methylation are important in the development of several cancers (4, 5). Some studies have revealed that DNA methylation occurs mainly at CpG islands, which are clustered by CG dinucleotides and found in gene promoters (6, 7). The promoter methylation of short stature homeobox gene two (*SHOX2*) and RAS association domain family 1, isoform A (*RASSF1A*) have been identified as diagnostic and prognostic biomarkers for lung cancer (8). A previous study has illustrated that *SHOX2* DNA methylation identified 62% of cytological negative cases (9). Another study has discovered that the sensitivities of these biomarkers on paired tumor and paracancerous samples were 77.78%, and the specificities were 100%. This suggests that these biomarkers could be useful as diagnostic biomarkers for early lung adenocarcinoma (LUAC) detection (10). Therefore, we aimed to explore the role of *SHOX2* and *RASSF1A* methylation in BFF/BALF for lung cancer diagnosis.

Our research innovatively combines with cytological and DNA methylation detection after bronchoscopic diagnosis, and we attempt to establish a new process for the diagnosis of lung cancer. At the same time, we hope that through this study, we can further clarify the clinical value of DNA methylation detection by bronchoscopic sampling in the auxiliary diagnosis of lung cancer. The diagnostic effectiveness of various diagnostic methods for visible and invisible findings by bronchoscopy was analyzed. In this study, we diagnosed 686 patients using bronchoscopy and detected aberrant methylation of the *SHOX2* and *RASSF1A* genes in BFF/BALF. Furthermore, a cytology examination of BFF/BALF was performed. Subsequently, we evaluated the diagnostic value of these three methods and their combinations, using clinical diagnosis as the gold standard.

## 2 Materials and methods

### 2.1 Study participants and samples

This study retrospectively analyzed data of 686 patients who underwent bronchoscopy at the Fourth Hospital of Hebei Medical University - Hebei Tumor Hospital between April 2020 and May 2022. Using the clinical diagnosis as the gold standard, we evaluated bronchoscopy, cytology of BALF, and aberrant methylation detection results. Among 686 patients, 585 were diagnosed with lung cancer: 181 with LUAC, 162 with LUSC, 135 with SCLC, 4 with large cell neuroendocrine carcinoma (LCNC), 4 with sarcomatoid carcinomas (SC), and 99 were undefined. The other 101 cases were controls, of which 92 were benign lung diseases and 9 were severe atypical hyperplasia (atypical epithelial hyperplasia). Of all patients with lung cancer, 43.9% were aged 61–70 years old, which is higher than the other age groups (including the ≤ 50, 51–60, and ≥ 71 years age groups). There was no significant difference in age composition between the lung cancer and control groups (P = 0.307). In the lung cancer group, 74% of patients were male, and 26% were female, with significant differences in sex composition (P = 0.01). **Table 1** summarizes the demographic and clinical features of the patients. Ethical review and approval as well as the need for patient consent were waived for this study due to: (1) All diagnostic and testing methods used in this article have been certified by the National Medical Products Administration of China and can be used in clinical diagnosis. (2) All the diagnoses and tests involved were routinely performed at the author’s hospital. (3) This study retrospectively analyzed data from patients diagnosed with lung diseases in the hospital over the past 2 years. Therefore, this study did not require an ethical certification or a signed informed consent form from patients.

**Table 1:** Demographic and clinical features of the patients.

	Lung Cancer		Control	
	n	%	n	%
Total	585	85.30%	101	14.70%
Age (years)				
≤50	44	7.50%	8	7.90%
51–60	145	24.80%	24	23.80%
61–70	257	43.90%	44	43.60%
≥71	138	23.60%	19	18.80%
Median age	64		63	
Age range	33–89		34–82	
Sex				
Male	433	74.00%	58	57.40%
Female	152	26.00%	43	42.60%
Histology subtype				
Lung Cancer				
LUAC	181	30.90%	-	-
LUSC	162	27.70%	-	-
SCLC	135	23.10%	-	-
LCNC	4	0.70%	-	-
SC	4	0.70%	-	-
Undefined	99	16.90%	-	-
Control				
Benign lung disease	-	-	92	91.10%
Severe atypical hyperplasia	-	-	9	8.90%

LUAC, Lung adenocarcinoma; LUSC, Lung squamous cell carcinoma; SCLC, Small cell lung cancer; LCNC, Large cell neuroendocrine carcinoma; SC, Sarcomatoid carcinoma.

### 2.2 Bronchoscopy

Bronchoscopy was performed according to the summary of the British Thoracic Society guidelines for diagnostic flexible bronchoscopy in adults in 2013 (11). The bronchoscope used was an Olympus CV-260SL model that can enter the main, segmental, and subsegmental bronchus. If a new lesion or blockage was observed during bronchoscopy, we flushed the visible findings and recovered the fluid, and defined it as BFF. If no visible findings were observed during bronchoscopy, we performed bronchoalveolar lavage and recovered the fluid, and defined it as BALF. The flushing operation and bronchoalveolar lavage were performed as follows.

We injected 40 mL of 37°C sterilized normal saline into the new lesion (BFF) or the lung segment of the suspected lesion (BALF). Immediately, we used negative pressure to recover the lavage solution, lavage three times, and ensured that the total amount of recovered BFF/BALF was not less than 50 mL. Half of the BFF/BALF was used for cytological diagnosis, while the other half was used for gene methylation detection.

### 2.3 Cytological and pathological analysis

The 10 ml BFF/BALF sample was centrifuged at 2000 rpm for 10 min, the supernatant was discarded, and the cells were re-suspended in 10–15 ml ultrapure water. The samples were centrifuged at 2000 rpm for 10 min. The cells were fixed on a glass slide and subjected to Papanicolaou staining. Experienced pathologists in our hospital evaluated all slides.

### 2.4 DNA extractions and bisulfite modification of genomic DNA

The DNA methylation level in BFF/BALF samples was evaluated using the Conformite Europeenne (CE) and National Medical Products Administration (NMPA) - approved Lung-Me™ analytical system (Tellgen Corporation, Shanghai, China). The Lung-Me™ analysis system includes four different kits: a DNA extraction kit, a DNA purification kit, a DNA quantification kit, and a DNA methylation polymerase chain reaction (PCR) kit.

A 10 ml BFF/BALF sample was centrifuged at 10,000 rpm to collect the cell pellet. Genomic DNA was extracted using DNA extraction kit (main ingredient is isothiocyanate). Genomic DNA was treated with sodium bisulfite to modify unmethylated cytosine to uracil. Each sample’s concentration was quantified using a Qubit 4.0 fluorometer. We used 200 ng of DNA for sodium bisulfite modification and 50 ng of modified DNA for PCR analysis. DNA samples were rapidly frozen in liquid nitrogen and stored at −80°C.

### 2.5 Methylation-specific real-time fluorescent PCR analysis

PCR amplification targeting sequences modified with sodium bisulfite were detected using TaqMan probes. Sequences of primers and probes were as follows: SHOX2 forward primer (5′-TTGTTTTTGGGTTCGGGTT-3′) and reverse primer (5′-CATAACGTAAACGCCTATACTCG-3′); RASSF1A forward primer (5′-CGGGGTTCGTTTTGTGGTTTC-3′) and reverse primer (5′-CCGATTAAATCCGTACTTCGC-3′). Probe for *RASSF1A* and *SHOX2* were (FAM-TCGCGTTTGTTAGCGTTTAAAGT-BHQ) and (VIC-ATCGAACAAACGAAACGAAAATTACC-BHQ), respectively. The reactions were performed in a total volume of 40 µL, containing 5 µL of bisulfite-modified DNA, 250 µM dNTP, 0.8 µl of each primer (10 µM), 1.5–3 mM MgCl_2_, 20 µL of 2 × Taq buffer (including dNTPs and Taq polymerase), and 13.4 µL of double distilled water (ddH_2_O). Further, this was performed in a thermocycler with the following cycling parameters: 95°C for 10 min; 45 cycles of 95°C for 30 s; specific annealing temperature: 58°C for 35 s, 72°C for 30 s; and a final extension step at 72°C for 8 min.

Sulfite was used to modify unmethylated cytosine bases to uracil bases, which were then converted to thymine bases during PCR amplification. However, methylated cytosine bases remained, so that methylated and unmethylated cytosine bases could be distinguished. Specific primers targeting sequences after bisulfite modification were designed for PCR. PCR amplification products were detected using Taq-Man probes (85 nM). A plasmid containing the corresponding sequence of methylated genes was used as the positive control and purified water as the negative control. The *β-actin gene* (forward primer: 5′-AAGATAGTGTTGTGGGTGTAGGT-3′; reverse primer: 5′-CCTACTTAATACACACTCCAAAAC-3′; probe: CY5-ACACCAACCTCATAACCTTATCACAC-BHQ) served as the internal control for quantifying the total DNA input. The baseline position was adjusted (Threshold = 10000; Noise band = 0.8) to obtain the Ct value of the fluorescence signal. The Ct value of β-actin gene signals should be between 18 and 32. When the amplification curve of *RASSF1A* DNA signals had a smooth S-shape and the ΔCt value was ≤ 12, the sample was termed positive for *RASSF1A* methylation. When the amplification curve of *SHOX2* signals showed a smooth S-shape and the ΔCt value was ≤ 9, the sample was termed positive for *SHOX2* methylation.

As a product that has passed the NMPA certification in China, the cut-off value is formulated based on the validation of 1000 clinical samples (600 cases of lung cancer and 400 cases of benign lesions); next, the ROC curve is drawn, the Youden index is calculated, and the highest specificity and sensitivity were obtained. The cut-off value is related to the specificity of each index for tumor. The cut-off value of an index with good specificity is high, and the cut-off value of an index with poor specificity is low. The specificity of RASSF1A is high, and the cut-off value is ΔCt ≤ 12. The specificity of SHOX2 is low, and the cut-off value is ΔCt ≤ 9.

### 2.6 Statistical analysis

Statistical analysis was performed using the SPSS 19.0 software package (SPSS Inc., Chicago, IL). A chi-squared test was used to analyze the methylation frequency of *SHOX2* and *RASSF1A* genes, as well as bronchoscopy and cytological examinations. The receiver operating characteristic curve (ROC) was used to calculate the area under the ROC curve (AUC) to evaluate the diagnostic effect. Statistical significance was set at a P value < 0.05.

## 3 Results

### 3.1 Relationship between age, sex and histological subtype of lung cancer

For the lung cancer and control groups, there was no significant difference in the age distribution of patients (P = 0.149). The sex-stratified analysis of patients with different pathological types (**Supplementary Table 1**) showed that more male than female patients had lung cancer (χ ^2 = ^29.335, P < 0.0001). Among them, the sex difference in patients with LUAC was insignificant (χ^2 = ^0.934, P = 0.334). Male patients had a significantly higher incidence of LUSC and SCLC than female patients (χ^2 = ^120.988, P < 0.0001 and χ^2 = ^35.267, P < 0.0001). Patients with LCNC and SC were all men. In the control group, the sex difference in benign lung disease was insignificant (χ^2 = ^0.696, P = 0.404). Severe atypical hyperplasia was significantly higher in male than in female patients (χ^2 = ^5.444, P = 0.02).

### 3.2 Location and histological classification of new lesions in bronchoscopy

**Figure 1:**
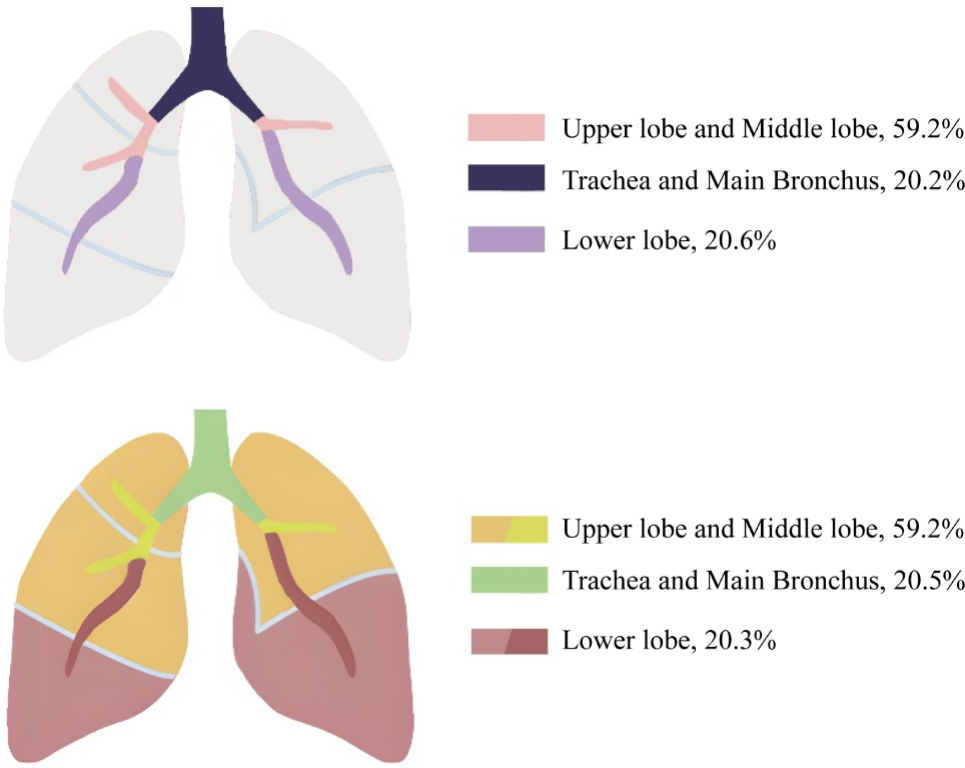
Distribution of new lesions in the lung and tumor location in the cancer group.

Among the 686 patients enrolled in this study, bronchoscopy revealed 392 cases of new lesions or blockages; 59.2% (232/392) of them had visible findings by bronchoscopy in the upper and middle lobes, 20.2% of them in the trachea and main bronchus, and 20.6% in the lower lobes. Among the 365 lung cancer patients with observable new lesions, 59.2% (216/365) had new lesions in the upper and middle lobes. If the right and left main bronchus were included, the proportion would rise to 79.7% (291/365). In addition, 81 patients had new lesions or blockages in their right and left lower lobes, with 91.4% (74/81) diagnosed with lung cancer (**Figure 1**; **Supplementary Table 2**). We performed histological classification of patients with bronchoscopy showing new lesions (**Supplementary Figure 1**). We discovered that LUAC mainly occurred in the right middle and left upper lobes; LUSC mainly occurred in the left and right upper lobes; SCLC mainly occurred in the right middle and right upper lobes; and two cases (50.0%) of LCNC occurred in the right lower lobe. The onset of two SC cases was in the right middle and left upper lobes. Approximately 294 patients were negatively diagnosed with bronchoscopy, of whom 220 were diagnosed with lung cancer. This indicates that although bronchoscopy can visually detect suspicious lesions, its diagnostic sensitivity remains insufficient.

Further analysis (**Table 2**) showed that the proportion of patients with visible findings by bronchoscopy among patients with LUAC was 31.5%, whereas the proportion of patients diagnosed with LUSC with visible new lesions or blockages by bronchoscopy was 87.0%. Furthermore, the proportion of patients with SCLC was 77.0%, which was significantly higher than that of patients with LUAC (P < 0.0001). Interestingly, in all histological subtypes of cancer, irrespective of the diagnostic result of bronchoscopy, the sensitivity of methylation detection was higher than that of cytology (P < 0.0001).

**Table 2:** Results of bronchoscopy in different histological subtype groups of lung cancer and sensitivity of DNA methylation in different types of each kind of histological subtype groups.

		Bronchoscopy		Cytological		*SHOX2* + *RASSF1A*	
		n	%	n (Pos.)	%	n (Pos.)	%
LUAC	Visible findings by bronchoscopy, BFF	57	31.5%	12	21.1%	38	66.7%
	No visible findings by bronchoscopy, BALF	124	68.5%	12	9.7%	57	46.0%
	Total	181		24	13.3%	95	52.5%
LUSC	Visible findings by bronchoscopy, BFF	141	87.0%	16	11.3%	121	85.8%
	No visible findings by bronchoscopy, BALF	21	13.0%	2	9.5%	13	61.9%
	Total	162		18	11.1%	134	82.7%
SCLC	Visible findings by bronchoscopy, BFF	104	77.0%	5	4.8%	102	98.1%
	No visible findings by bronchoscopy, BALF	31	23.0%	3	9.7%	28	90.3%
	Total	135		8	5.9%	130	96.3%
LCNC	Visible findings by bronchoscopy, BFF	3	75.0%	0	0.0%	2	66.7%
	No visible findings by bronchoscopy, BALF	1	25.0%	1	100.0%	1	100.0%
	Total	4		1	25.0%	3	75.0%
SC	Visible findings by bronchoscopy, BFF	2	50.0%	1	25.0%	2	100.0%
	No visible findings by bronchoscopy, BALF	2	50.0%	0	0.0%	2	100.0%
	Total	4		1	25.0%	4	100.0%

SHOX2, short-stature homeobox gene two; RASSF1A, RAS association domain family 1, isoform A; LUAC, lung adenocarcinoma; LUSC, lung squamous cell carcinoma; SCLC, small cell lung cancer; LCNC, large cell neuroendocrine carcinoma; SC, sarcomatoid carcinoma; BFF, bronchial flushing fluid; BALF, bronchoalveolar lavage fluid.

### 3.3 Detection sensitivity of cytology and methylation in different histological subtype groups

We analyzed the diagnostic sensitivity of *SHOX2* and *RASSF1A* methylation in BFF/BALF in different histological subtypes of lung cancer (**Table 3**). The results showed that the sensitivity of *SHOX2* and *RASSF1A* methylation detection in the lung cancer and control groups was 72.8% and 12.9%, respectively. In addition, the sensitivity of cytological diagnosis in the lung cancer group was 10.4%, which was significantly lower than that of BFF/BALF methylation detection (P < 0.0001). In a more detailed analysis, we observed that the sensitivity of *SHOX2* and *RASSF1A* methylation in SCLC was 96.3%, whereas the sensitivity of cytology was 5.9%. Moreover, for patients with negative cytology, the PPV of SHOX2 and RASSF1A methylation was 96.3%. In addition, the results from the other four histological types showed similar performance (LUAC 52.5% vs. 13.3%, LUSC 82.7% vs. 11.1%, SCLC 96.3% vs. 5.9%, and undefined 60.6% vs. 9.1%), indicating that *SHOX2* and *RASSF1A* methylation in BFF/BALF is a genetic biomarker that can be used in almost all histological subtypes of lung cancer. By comparing the sensitivity of SHOX2 and RASSF1A, we found that in patients with LUAC, LUSC, LCNC, SC, and undefined, the sensitivity of SHOX2 methylation detection was significantly higher than that of RASSF1A (P = 0.012), and in patients with SCLC, the sensitivity of RASSF1A methylation was higher (88.1% vs. 79.3%). However, the number of LCNC and SC cases was insufficient to represent this histological subtype (n = 4), which can be studied further.

**Table 3:** Detection sensitivity of cytology and *SHOX2* and *RASSF1A* methylation panel in different histological subtype groups.

Histology subtype	Total	Cytology		SHOX2		RASSF1A		SHOX2 + RASSF1A		SHOX2 + RASSF1A + Cytology	
		n	Sensitivity	n	Sensitivity	n	Sensitivity	n	Sensitivity	n	Sensitivity
Lung Cancer											
LUAC	181	24	13.3%	82	45.3%	51	28.2%	95	52.5%	101	55.8%
LUSC	162	18	11.1%	131	80.9%	41	25.3%	134	82.7%	136	84.0%
SCLC	135	8	5.9%	107	79.3%	119	88.1%	130	96.3%	130	96.3%
LCNC	4	1	25.0%	3	75.0%	2	50.0%	3	75.0%	3	75.0%
SC	4	1	25.0%	4	100.0%	2	50.0%	4	100.0%	4	100.0%
Undefined	99	9	9.1%	50	50.5%	40	40.4%	60	60.6%	63	63.6%
Total	585	61	10.4%	377	64.4%	255	43.6%	426	72.8%	437	74.7%
Control											
Benign lung disease	92	0	0.0%	5	5.4%	5	5.4%	8	8.7%	8	8.7%
Severe atypical hyperplasia	9	0	0.0%	5	55.6%	1	11.1%	5	55.6%	5	55.6%
Total	101	0	0.0%	10	9.9%	6	5.9%	13	12.9%	13	12.9%

LUAC, Lung adenocarcinoma; LUSC, Lung squamous cell carcinoma; SCLC, Small cell lung cancer; LCNC, Large cell neuroendocrine carcinoma; SC, Sarcomatoid carcinoma.

The cytologic evaluation may be confusing to the clinicians. Further analysis of our data revealed that 42.7% (293/686) of all the enrolled patients were diagnosed as cytologically undetermined. Among these patients, 272 were finally diagnosed with lung cancer, of which 84.6% (230/272) were methylation positive, and 21 were diagnosed with benign diseases with 23.8% as methylation positive. Among 585 patients with lung cancer, 61 were with positive cytology, of which 82.0% (50/61) were methylation positive; 272 cases were with undetermined cytology, of which 84.6% were methylation positive; 252 cases were with negative cytology, of which 57.9% were methylation positive (**Supplementary Figure 2**). The data above suggested that *SHOX2* and *RASSF1A* methylation panel in BFF/BALF could improve the sensitivity of cytological diagnosis and reduce uncertain diagnostic results.

**Figure 2:**
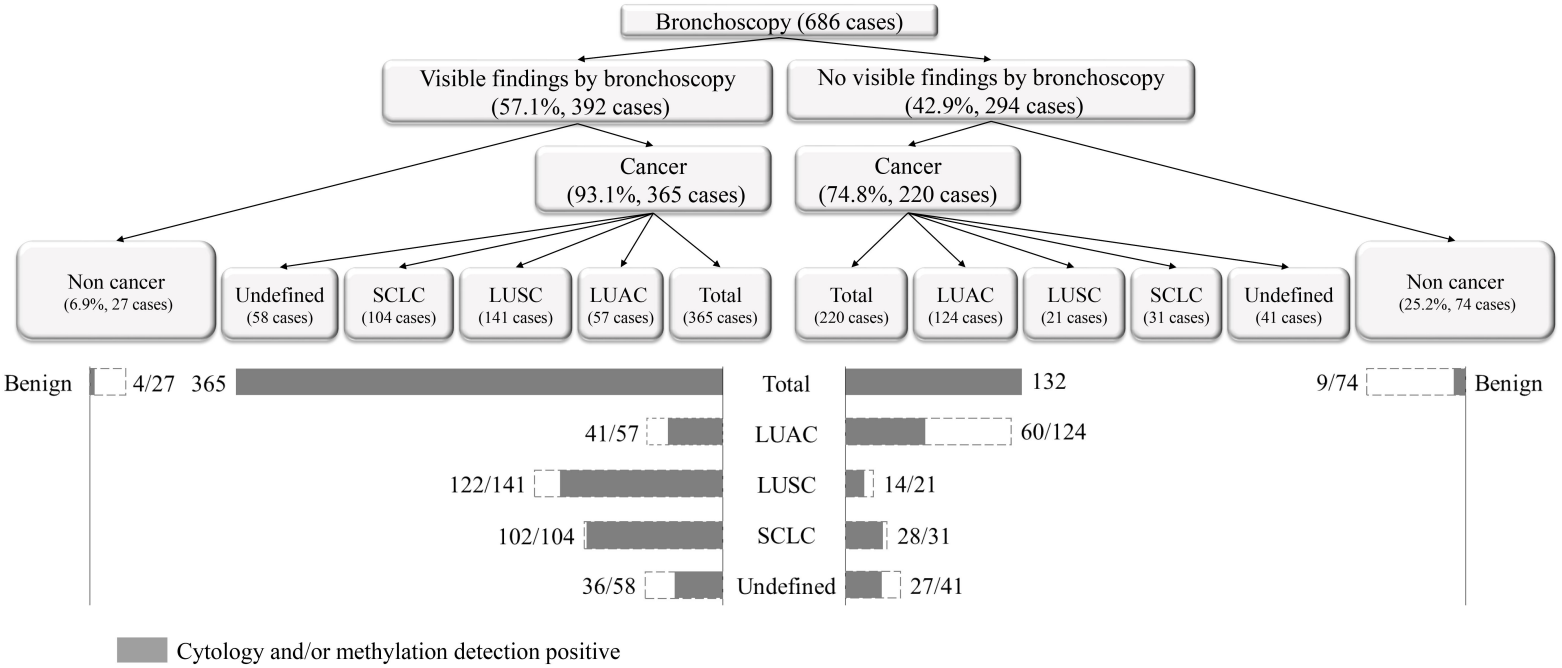
The comprehensive diagnostic process and result of morphological combined molecular methylation under bronchoscopy.

According to the comprehensive diagnostic process and result shown in **Figure 2**, 57.1% of the patients showed visible findings by bronchoscopy, 93.1% of whom were diagnosed with lung cancer. The proportion of patients without visible findings was 42.9%, of which 74.8% were diagnosed with lung cancer. In patients with LUSC and SCLC, lung cancer patients with visible findings by bronchoscopy is higher than those without visible findings (122 vs. 14 and 102 vs. 28), while that of LUAC is the opposite (41 vs. 60).

### 3.4 ROC curve analysis of the *SHOX2* and *RASSF1A* methylation panel in BALF

**Figure 3:**
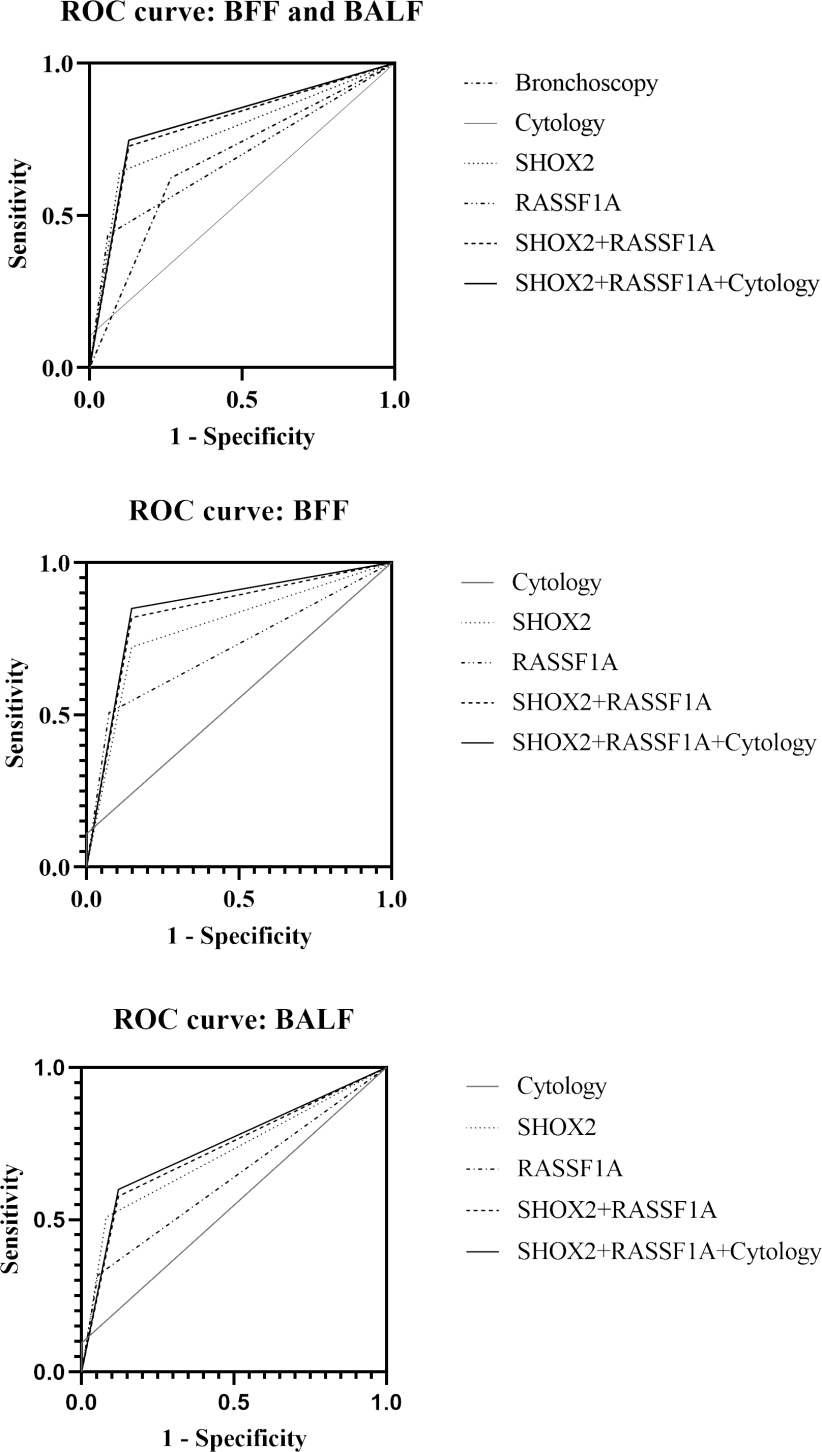
Receiver operating characteristic curve of bronchoscopy, cytology, and *SHOX2* and *RASSF1A* methylation panel.

We compared the diagnostic efficacy of bronchoscopy, cytology, and methylation detection using ROC curve analysis (**Figure 3**). Compared with bronchoscopy (AUC value: 0.678, 95% CI: 0.6231–0.7335) and cytological diagnosis (AUC value: 0.552, 95% CI: 0.4956–0.6086), *SHOX2* and *RASSF1A* methylation detection in BFF/BALF had the highest diagnostic efficacy, with an AUC value of 0.800 (95% CI: 0.7558–0.8437) (**Figure 3** and **Table 4**). Moreover, methylation analysis of the *SHOX2* and *RASSF1A* panel showed a higher diagnostic sensitivity of 72.8%, compared to bronchoscopy (62.4%), cytology (10.4%), single *SHOX2* methylation (64.3%), and single *RASSF1A* methylation (43.6%). Notably, when the *SHOX2* and *RASSF1A* methylation panel was combined with cytology, the AUC improved to 0.809 (95% CI: 0.7656–0.8527), and the sensitivity and specificity were 74.7% and 87.1%, respectively, suggesting that *SHOX2* and *RASSF1A* methylation detection of BFF/BALF could be an effective complementary tool of cytology in lung cancer diagnosis.

**Table 4:** The diagnostic efficacy of bronchoscopy, cytology, *SHOX2* and *RASSF1A* methylation panel.

	AUC		Sensitivity	Specificity	PPV	NPV
	Value	95% CI				
BFF and BALF						
Bronchoscopy	0.678	0.6231–0.7335	62.4%	73.3%	94.3%	25.2%
Cytology	0.552	0.4956–0.6086	10.4%	100.0%	100.0%	16.2%
* SHOX2*	0.772	0.7284–0.8153	64.3%	90.1%	98.7%	30.3%
* RASSF1A*	0.688	0.6414–0.7351	43.6%	94.1%	98.1%	22.4%
* SHOX2* + *RASSF1A*	0.800	0.7558–0.8437	72.8%	87.1%	98.2%	35.6%
* SHOX2* + *RASSF1A* + Cytology	0.809	0.7656–0.8527	74.7%	87.1%	95.2%	40.4%
BFF						
Cytology	0.555	0.0815-0.1458	11.0%	100.0%	100.0%	7.7%
* SHOX2*	0.788	0.6752-0.7667	72.3%	85.2%	98.5%	18.5%
* RASSF1A*	0.716	0.4558-0.5578	50.7%	92.6%	98.9%	13.9%
* SHOX2* + *RASSF1A*	0.836	0.7764-0.8553	81.9%	85.2%	97.7%	25.8%
* SHOX2* + *RASSF1A* + Cytology	0.851	0.8090-0.8824	84.9%	85.2%	99.0%	30.4%
BALF						
Cytology	0.548	0.0632-0.1415	9.5%	100.0%	100.0%	27.1%
* SHOX2*	0.714	0.4434-0.5744	50.9%	91.9%	94.9%	38.6%
* RASSF1A*	0.632	0.2602-0.3824	31.8%	94.6%	94.6%	31.8%
* SHOX2* + *RASSF1A*	0.728	0.5112-0.6407	57.7%	87.8%	93.3%	41.1%
* SHOX2* + *RASSF1A* + Cytology	0.739	0.5341-0.6625	60.0%	87.8%	93.6%	42.5%

AUC, area under the curve, CI, confidence interval, PPV, positive predictive value, NPV, negative predictive value.

In addition, BFF samples and BALF samples were analyzed separately. The results showed that the AUC value of methylation detection was higher than that of cytology (BFF, 0.836 vs. 0.555; BALF, 0.728 vs. 0.528). When methylation and cytology were combined for diagnosis, the PPV of BFF sample was 99.0%, and the NPV of BALF sample was 42.5%.

### 3.5 Case sharing – methylation detection provides key evidence for lung cancer surgery determination

The patient is a married 56-year-old man with a smoking history of 60 pack-years. The patient was admitted to the hospital because of back pain and occasional cough for 3 months. The patient has no history of hypertension, diabetes, coronary heart disease, infectious diseases such as tuberculosis and hepatitis, surgery, or blood transfusion.

**Figure 4:**
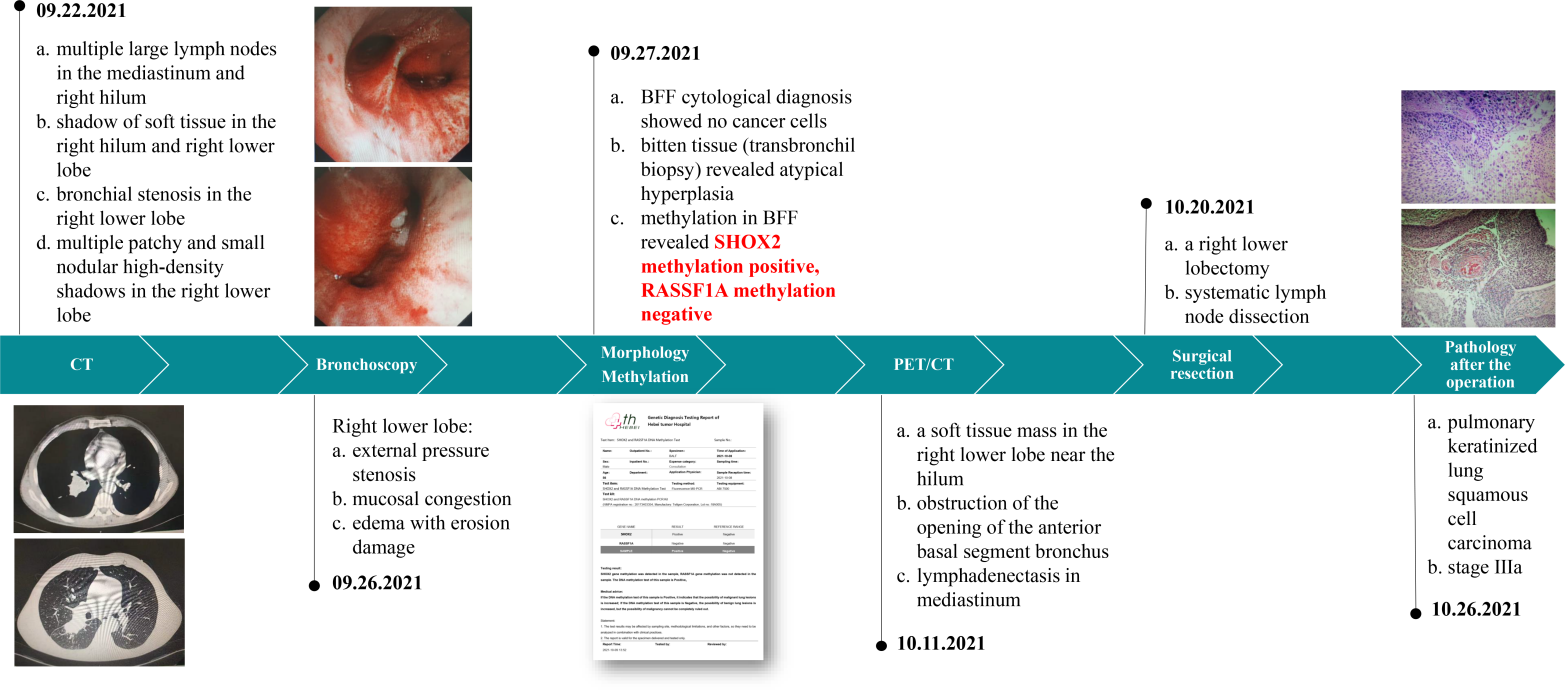
Patient medical history.

The medical history of patients in our hospital is shown in **Figure 4**.

## 4 Discussion

Previous studies have revealed a relationship between cancer diagnosis and the detection of aberrant methylation changes in the *SHOX2* and *RASSF1A* genes (12–14). These studies have demonstrated that detecting *SHOX2* and *RASSF1A* methylation has a good complementary and prompt effect on the cytological diagnosis of lung cancer. BFF/BALF is a noninvasive specimen obtained easily via fiberoptic bronchoscopy and is a routine examination for patients with suspected lung cancer. Furthermore, BFF/BALF is an alternative source of genetic diagnostic markers because it is derived from tumors or surrounding tissues. In laboratory work, the obtained BFF/BALF is generally used for methylation detection and cytological diagnosis at the same time. The BFF/BALF recovered from patients will be homogenized, and 10 mL of the sample will be sub-packed into cell preservation tubes. Next, 25 mL of the BFF/BALF sample will be used for cytological diagnosis. The sample volume required for methylation detection is less than that required for cytology. Generally, the range of cell concentration in the collected BFF/BALF is relatively broad. Before the methylation detection experiment, the DNA concentration of each sample will be measured. Only samples with DNA concentration ≥ 1 ng/µL can be used for subsequent experiments, and the added DNA quality is specified to be 20 ng during sulfite modification. When the concentration cannot reach 20 ng, all the extracted DNA will be used for PCR experiment. In actual clinical work, 99% of the DNA concentration of BFF/BALF samples can meet the needs of methylation detection. The above operations can play a quality control role in the methylation detection experiment and effectively ensure the stability of the experiment.

DNA methylation has been proven as a milestone in tumorigenesis, and the detection of abnormal methylation of some genes, including *SHOX2* and *SEPTIN9*, has been converted into commercial kits (15). We used human *SHOX2* and *RASSF1A* gene methylation detection kits (Tellgen Co. Ltd., Shanghai, China) to detect the methylation status of 686 patients. This kit has high sensitivity and specificity, a closed reaction system, detection and analysis integration, automation, and high throughput (16, 17). In addition, the technical platform of this study was based on multiplex real-time fluorescence PCR. Multiple fluorescence channels can detect *RASSF1A*, *SHOX2*, and *β-actin* simultaneously. Aside from saving labor and time, the more important reason was that *β-actin*, as the internal control, should be detected in the same tube to facilitate the monitoring of the entire PCR process.

The accuracy of bronchoscopy and sampling is the basis of subsequent cytological diagnosis and methylation detection, and the accuracy of sampling has an impact on the diagnostic results. Our study used the stratified analyzed sampling methods, including accurate sampling method (BFF) and distal alveolar lavage (BALF). Different hospitals implement different bronchoscopic techniques. Hospitals with good equipment tend to have more BFF samples, and hospitals with poor equipment tend to have more BALF samples. The methylation detection sensitivity of BFF samples and BALF samples was significantly different (P < 0.001). Furthermore, we compared the diagnostic value of bronchoscopy in different histological subtypes of lung cancer. Bronchoscopy sensitivity was higher in SCLC and LUSC than in LUAC. Central bronchogenic carcinoma occurs in the main and segmental bronchus, which is difficult to diagnose in the early stages. In future studies, we intend to detect cell methylation in the sputum for auxiliary diagnosis. During sputum expectoration, the upper lobe, middle lobe, and main bronchus may be the first to be mobilized, followed by the oral cavity.

Our data showed that the sensitivity of *SHOX2* and *RASSF1A* methylation in lung cancer diagnosis was 72.8%, the specificity was 87.1%, and the AUC value was 0.800. This finding indicates that detecting *SHOX2* and *RASSF1A* methylation has good diagnostic efficacy in lung cancer diagnosis. In all histological subtypes, the performance of the combined detection of methylation was satisfactory. The sensitivity of the panel in LUAC, LUSC, SCLC, LCNC, and SC were 52.5%, 82.7%, 96.3%, 75.0%, and 100.0%, respectively. Compared with LUAC, the *SHOX2* and *RASSF1A* methylation panel performed better in SCLC and LUSC, similar to bronchoscopy in this study and previous studies (8, 18). Furthermore, DNA methylation in BFF had higher positive rates than in BALF. A probable explanation is that most LUSC and SCLC are central bronchogenic carcinomas with visible endoscopic findings. However, some peripheral lung cancers grow inward and break through the tracheal wall, becoming peripheral lung cancer with visible endoscopic findings, including a part of peripheral LUAC and LUSC. Therefore, BFF obtained from the visible findings of bronchoscopy has high diagnostic efficacy in lung cancer with visible endoscopic findings. Besides, the coincidence rate between cytology positive and methylation positive results was 82%. Methylation detection can provide more evidence for clinicians. Patients with cytology undetermined diagnosis but methylation positive are more likely to have malignant tumors. Therefore, joint detection can effectively reduce uncertain diagnostic results.

This study analyzed BALF/BFF from 297 patients with LUSC and SCLC, including 253 men and 44 women. One possible explanation is that the male smoking population is significantly higher than the female population, increasing the central lung cancer incidence (19). In addition, the patients in this study were all residents of Hebei Province, China, the second largest iron mine-producing province in China and one of China’s 13 coal mine bases. Some studies suggest that occupational exposure to coal and metal mines can increase SCLC incidence (20). Methylation analysis of *SHOX2* and *RASSF1A* panels showed a sensitivity of 96.3% compared to cytology (5.9%). Additionally, we discovered that the *SHOX2* and *RASSF1A* methylation panel in BALF/BFF has a high AUC value of 0.917 compared to cytology. SCLC is a lung cancer with rapid growth and high mortality. The cytological diagnosis of SCLC is relatively difficult because in the actual work of the pathology department, the difficulty of diagnosis of small cell carcinoma is generally attributed to the following two points: (1) most SCLC cells are small in size and similar with lymphocytes; (2) when diagnosing samples such as BFF/BALF, the number of cancer cells is small. If there is no obvious aggregation and adhesion, it is difficult to directly determine them as cancer cells. In the future, in-depth studies can be conducted by combining the history of smoking, exposure to risk factors, tumor immune markers, and immunohistochemistry with methylation detection to improve the sensitivity of the early diagnosis of SCLC.

In our study, nine cases of severe atypical hyperplasia were assigned to the control group because these patients did not have a precise diagnosis of malignancy. Among them, three patients showed new lesions or blockages during bronchoscopy, and the methylation test was positive, suggesting that clinical doctors should pay more attention to these patients (**Supplementary Figure 3**). Dysplasia and carcinoma *in situ* are precancerous lesions with diverse morphological manifestations and poor diagnostic consistency. Methylation detection is more objective and has a high cancer specificity. *SHOX2* and *RASSF1A* methylation has been associated with high expression of Ki-67, which may indicate that cancer cells are proliferating (10). In addition, the cytological diagnosis of BALF in all severe atypical hyperplasia cases was negative, whereas five patients tested positive for methylation. This indicates that methylation tests may facilitate risk triage for patients who cannot be diagnosed using cytology and help clinical physicians formulate reasonable follow-up strategies. However, owing to the small sample size and the lack of continuous follow-up, the diagnostic efficacy of methylation detection in patients with severe atypical hyperplasia still needs further study.

The relationship between methylation detection and tumor size, gene mutation, and patient prognosis was not studied in this article. In addition, bronchoscopy biopsy samples are the most sensitive materials for cytological diagnosis among all samples taken under bronchoscope, but they can only be obtained under the condition of visible changes, and bronchoscopy biopsy is also affected by the location of samples, tumor size, and necrotic tissue. BALF/BFF is easier to obtain, and combined with the high sensitivity of methylation detection, it can be used to identify more of early lung cancer patients. At the same time, to ensure the accuracy of the study, it is necessary to use the same type of samples to compare the diagnostic effectiveness between different methodologies. Therefore, in this study, we only analyzed and discussed the diagnostic efficacy of cytology and methylation detection of BALF/BFF samples and did not analyze the biopsy samples. In future studies, we will try to explore the application value of methylation detection of bronchial biopsy samples in clinical diagnosis and prognosis evaluation.

We hope that this discussion of morphological and molecular methylation under bronchoscopy will be useful in clinical setting. This assay also has potential clinical usefulness outside of routine bronchoscopy, such as for patients with peripheral lesions (where diagnostic yield of biopsy is lower or with negative initial cytology) or patients in resource-poor situations/regions where access to advanced diagnostic bronchoscopy (EBUS, electromagnetic navigation, robotic navigation, etc.) and/or percutaneous biopsy are limited. In conclusion, *SHOX2* and *RASSF1A* methylation detection in BFF/BALF can be used to diagnose lung cancer. Therefore, methylated *SHOX2* and *RASSF1A* genes may be promising tumor markers in lung cancer diagnosis. Methylation detection may serve as a supplementary tool for cytological diagnosis, and its combination with bronchoscopy may provide a more effective diagnostic process.

## Data availability statement

The original contributions presented in the study are included in the article/**Supplementary Material**, further inquiries can be directed to the corresponding author/s.

## Ethics statement

Ethical review and approval was not required for the study on human participants in accordance with the local legislation and institutional requirements. Written informed consent for participation was not required for this study in accordance with the national legislation and the institutional requirements. Written informed consent was obtained from the individual(s) for the publication of any potentially identifiable images or data included in this article.

## Author contributions

Conceptualization, JZ, HH, and ZS. Methodology, JZ, HH, and BS. Resources, FY and RW. Data curation, YB, HL, and SK. All authors contributed to the article and approved the submitted version.
