# Supplementary material for: A comprehensive diagnostic scheme of morphological combined molecular methylation under bronchoscopy
Source: Front Oncol. 2023 Apr 27;13:1133675. doi: 10.3389/fonc.2023.1133675 (PMC10174301; doi:10.3389/fonc.2023.1133675)
Supplement: Supplementary file 1 [file DataSheet_1.docx]

Supplementary Material

A Comprehensive Diagnostic Scheme of Morphological Combined Molecular Methylation Under Bronchoscopy

Jinze Zhang, Haoran Huang, Fan Yu, Yuanyuan Bian, Rui Wang, Hui Liu, Saisai Kang, Bin She, Zhihua Shi^*^

*** Correspondence:** Zhihua Shi: shibukedang2000@163.com

# Supplementary Figures and Tables

## Supplementary Figures


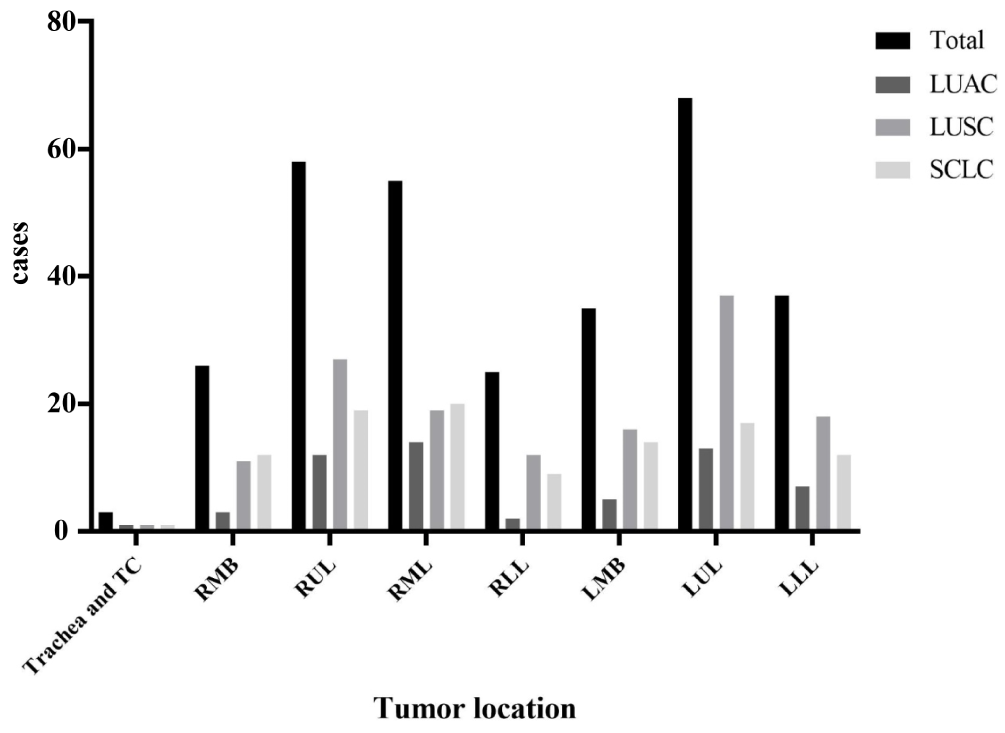


**Supplementary Figure 1**. Relationship between tumor location and histological subtype in lung cancer. Abbreviations: TC, trachea carina; RMB, right main bronchus; RUL, right upper lobe; RML, right middle lobe; RLL, right lower lobe; LMB, left main bronchus; LUL, left upper lobe; LLL, left lower lobe.


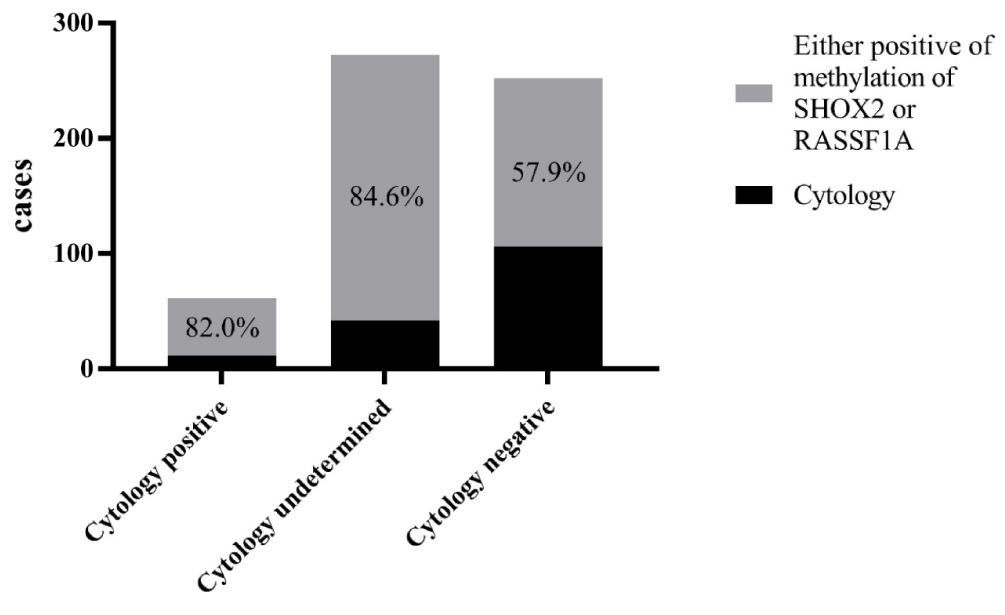


**Supplementary Figure 2**. Sensitivity of methylation detection in different cytological results of the lung cancer group


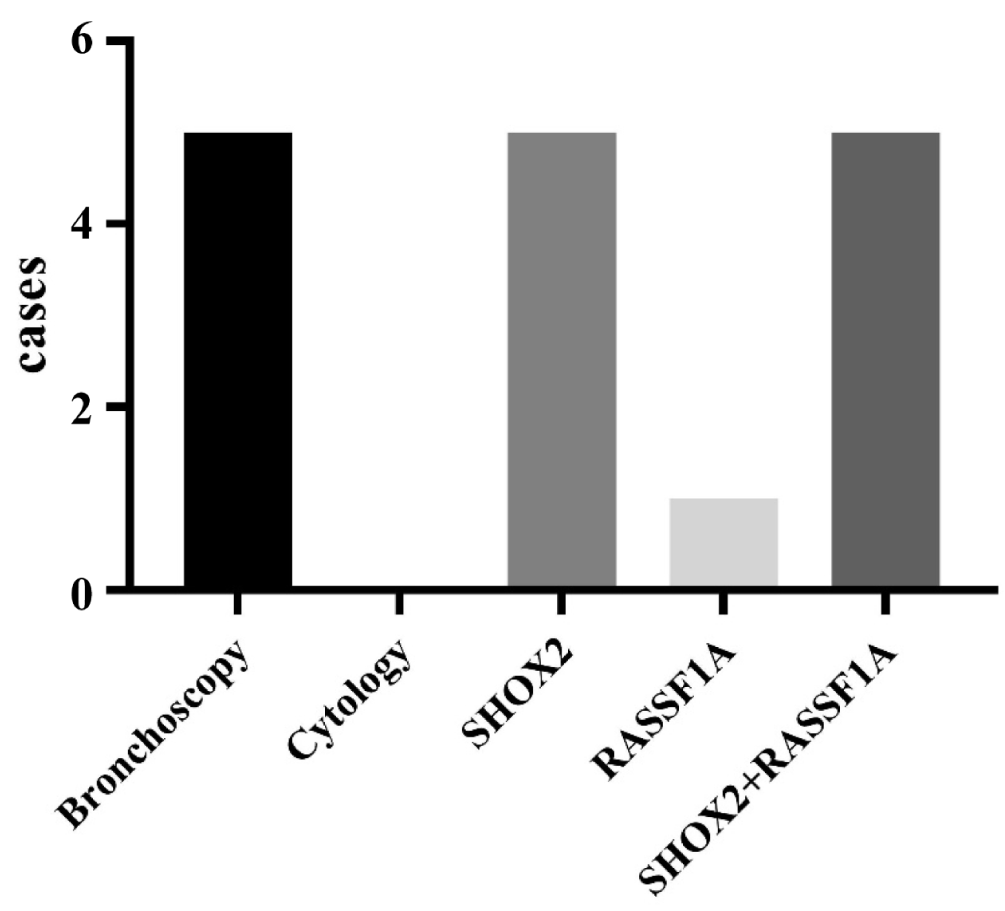


**Supplementary Figure 3**. Number of positive patients in different diagnostic methods with severe atypical hyperplasia

## Supplementary Tables

**Supplementary Table 1**. Relationship between sex and histological subtypes of lung cancer

| Histology subtype | Total | Male | | Female | |
| --- | --- | --- | --- | --- | --- |
|  |  | n | % | n | % |
| Lung Cancer | 585 | 358 | 61.2% | 227 | 38.8% |
| LUAC | 181 | 97 | 53.6% | 84 | 46.4% |
| LUSC | 162 | 151 | 93.2% | 11 | 6.8% |
| SCLC | 135 | 102 | 75.6% | 33 | 24.4% |
| LCNC | 4 | 4 | 100.0% | 0 | 0.0% |
| SC | 4 | 4 | 100.0% | 0 | 0.0% |
| Control | 101 | 58 | 57.4% | 43 | 42.6% |
| Benign lung disease | 92 | 50 | 54.3% | 42 | 45.7% |
| Severe atypical  hyperplasia | 9 | 8 | 88.9% | 1 | 11.1% |

**Supplementary Table 2.** Location of new lesions

|  | | Total | | Lung Cancer | | Control | |
| --- | --- | --- | --- | --- | --- | --- | --- |
|  |  | n | % | n | % | n | % |
| New lesion |  | 392 |  | 365 | 93.1% | 27 | 6.9% |
| UL and ML | Total | 232 | 59.0% | 216 | 59.2% | 16 | 59.3% |
|  | RUL | 75 | 19.1% | 74 | 20.3% | 1 | 3.7% |
|  | RML | 70 | 17.8% | 61 | 16.7% | 9 | 33.3% |
|  | LUL | 87 | 22.1% | 81 | 22.2% | 6 | 22.2% |
| LL | Total | 81 | 20.6% | 74 | 20.3% | 7 | 25.9% |
|  | RLL | 33 | 8.4% | 31 | 8.5% | 2 | 7.4% |
|  | LLL | 48 | 12.2% | 43 | 11.8% | 5 | 18.5% |
| Trachea and MB | Total | 79 | 20.1% | 75 | 20.5% | 4 | 14.8% |
|  | Trachea and TC | 3 | 0.8% | 3 | 0.8% | 0 | 0.0% |
|  | RMB | 34 | 8.7% | 31 | 8.5% | 3 | 11.1% |
|  | LMB | 42 | 10.7% | 41 | 11.2% | 1 | 3.7% |
| Normal |  | 294 |  | 220 | 74.8% | 74 | 25.2% |

Abbreviations: UL, Upper lobe; ML, Middle lobe; LL, Lower lobe; LUL, Left upper lobe; LLL, Left lower lobe; RUL, Right upper lobe; RML, Right middle lobe; RLL, Right lower lobe; TC, Trachea carina; MB, Main bronchus; RMB, Right main bronchus; LMB, Left main bronchus
